# New Azido Coumarins as Potential Agents for Fluorescent Labeling and Their “Click” Chemistry Reactions for the Conjugation with *closo*-Dodecaborate Anion

**DOI:** 10.3390/molecules27238575

**Published:** 2022-12-05

**Authors:** Julia Laskova, Alexander Serdyukov, Irina Kosenko, Ivan Ananyev, Ekaterina Titova, Anna Druzina, Igor Sivaev, Anastasia A. Antonets, Alexey A. Nazarov, Vladimir I. Bregadze

**Affiliations:** 1 A.N. Nesmeyanov Institute of Organoelement Compounds, Russian Academy of Sciences, 28 Vavilov Str., 119334 Moscow, Russia; 2 M.V. Lomonosov Institute of Fine Chemical Technology, MIREA—Technological University, 86 Vernadsky Avenue, 119571 Moscow, Russia; 3 N.S. Kurnakov Institute of General and Inorganic Chemistry, Russian Academy of Sciences, 31 Leninsky Avenue, 119991 Moscow, Russia; 4 Basic Department of Chemistry of Innovative Materials and Technologies, G.V. Plekhanov Russian University of Economics, 36 Stremyannyi Line, 117997 Moscow, Russia; 5 Department of Chemistry, M.V. Lomonosov Moscow State University, Leninskie Gory 1/3, 119991 Moscow, Russia

**Keywords:** coumarins, azido derivatives, closo-dodecaborate, “click” reaction, fluorescent labeling, cellular uptake

## Abstract

Novel fluorescent 7-methoxy- and 7-(diethylamino)-coumarins modified with azido-group on the side chain have been synthesized. Their photophysical properties and single crystals structure characteristics have been studied. In order to demonstrate the possibilities of fluorescent labeling, obtained coumarins have been tested with *closo*-dodecaborate derivative bearing terminal alkynyl group. Cu^I^ catalyzed Huisgen 1,3-dipolar cycloaddition reaction has led to fluorescent conjugates formation. The absorption–emission spectra of the formed conjugates have been presented. The antiproliferative activity and uptake of compounds against several human cell lines were evaluated.

## 1. Introduction

Coumarins are an important class of natural products that exhibits various types of biological activity, such as anti-inflammatory [1,2,3], anticonvulsant [4], antibacterial [5,6,7], antiviral [8,9,10], anticancer [11,12,13,14,15] and others [16,17,18,19,20,21,22,23,24,25,26,27,28,29]. Along with biological activity, coumarins have been known to possess unique fluorescent properties, especially when substituted with an electron donating group at the C-7 and an electron withdrawing group at the C-3 position of coumarin moiety. Such coumarins have been namely characterized with large Stokes shifts, high quantum yields of fluorescence, and minimal fluorophore degradation, which resulted to their wide use in medicinal chemistry and various biological study as fluorescent probes and labels [30,31,32,33].

**Figure 1:**
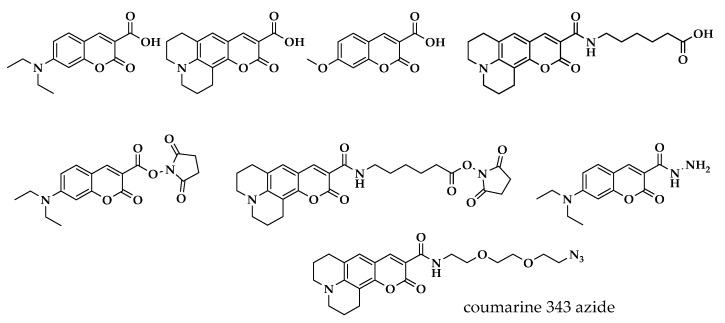
Some commercially available fluorescent coumarins.

Some commercially available and commonly used for labeling fluorescent coumarins are shown in Figure 1. Functionalized with carboxylic groups or with activated esters, coumarins (Figure 1) have been aimed for detection of amino acids, small sized proteins, and antibodies through the peptide bond formation [34,35,36,37,38,39,40,41,42,43]. 7-Diethylamino-coumarin-3-carbohydrazide is used for fluorescence labeling of carbonylated proteins and lipids [44,45,46,47,48].

Since the discovery by Sharpless the term of “click chemistry” for the [3+2] cycloaddition reactions of alkynes with organic azides, which are high-yielding, practical, operationally safe, avoid by-product formation, and proceed in environment friendly solvents at room temperature and generally under mild conditions [49,50], this approach is widely used for synthesis of various bioactive coumarin derivatives [51,52], as well as fluorescent labeling of nanoparticles and biomolecules using azido derivatives of coumarin, such as coumarin 343 azide (Figure 1) [53,54,55].

However, in some cases labelling requires the use of coumarin azides with a shorter spacer between the fluorescent part and the azide function. Herein, we report the synthesis, fluorescence properties, and crystal structures of two new azido derivatives of coumarin and their use for labelling of the *closo*-dodecaborate anion, including the evaluation of antiproliferative activity and uptake of the synthesized compounds against several human cell lines.

## 2. Results and Discussion

### 2.1. Strategy

The sodium salt of the mercapto derivative of the *closo*-dodecaborate anion Na_2_[B_12_H_11_SH] (BSH) is one of two clinically approved agents for boron neutron capture therapy (BNCT) for cancer [56,57,58], which is an anti-cancer treatment based on the irradiation of ^10^B-rich tumours with low energy neutrons. The neutron capture reaction produces high linear energy transfer particles, ^4^He (α-particle) and ^7^Li, which cause lethal damage to tumour cells through ionization process [59,60,61,62]. This causes increased interest in the synthesis of conjugates of the *closo*-dodecaborate anion with various biologically active molecules [57,58,63,64,65,66].

An important step in the evaluation of new agents for BNCT is to investigate their uptake in cells in vitro or in larger organisms in vivo. Molecular imaging using optical or radionuclide tags is ideal for small animal and human imaging. However, the use of radionuclide labels [67,68,69,70] is generally limited by the availability of appropriate radionuclides and the necessary special equipment for working with radioactive substances. Therefore, various fluorescent labels, such as *meso*-(4-hydroxyphenyl)-4,4-difluoro- 4-bora-3*a*,4*a*-diaza-*s*-indacene (BODIPY) [71], coumarin [72,73,74], and (3,6-diamino-9-(2,5- dicarboxyphenyl)-4,5-disulfo-Xanthylium) (Alexa Flour 488^®^) [75], are commonly used to label the *closo*-dodecaborate anion and its derivatives.

An effective approach to the functionalization of the *closo*-dodecaborate anion is the opening of its cyclic oxonium derivatives by nucleophiles [76]. This approach is widely used for the synthesis of its various functional derivatives and attachment to biomolecules [64,77,78], and has also been used to modify other polyhedral boron hydrides such as the *closo*-decaborate anion [64], *nido*-carborane [79], and cobalt and iron bis(dicarbollides) [80]. In particular, the synthesis of the earlier described coumarin derivatives of the *closo*-dodecaborate anion is based on the opening of oxonium rings with hydroxy groups at various positions of the coumarin heterocycle [72,73,74]. This leads to a significant difference in the photophysical characteristics of the obtained compounds from the commonly used fluorescent labels (see above). Therefore, our goal was to preserve the structure of coumarins traditionally used for labeling. We considered it expedient to use the [3+2]-cycloaddition reaction for the synthesis of coumarin derivatives of the *closo*-dodecaborate anion. The “click” chemistry reaction has found a wide synthetic application for the preparation of a wide range of conjugates based on the boron cluster [81,82,83,84,85,86,87,88,89,90]. However, the opening of the 1,4-dioxane derivative results in moieties where the boron cluster connected with functional fragment via a biologically compatible flexible di(ethylene glycol) spacer. Thus, in particular, derivatives of the *closo*-dodecaborate anion with a terminal alkynyl group were obtained [91,92]. Therefore, the use of commercially available coumarin 343 azide will result in a product with an unnecessarily long spacer between the boron cluster and the fluorescent label.

Taking into account these limitations, we decided to modify the known 7-diethylamino- and 7-methoxy-coumarin-3-carboxylic acids [40,41] by forming amides with 2-azidoethylamine.

### 2.2. Synthesis

**Scheme 1:**
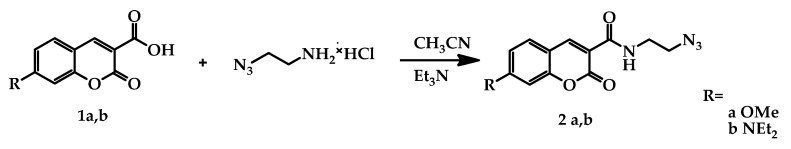
Synthesis of coumarins **2a,b**.

The target coumarins functionalized with azido group **2a,b** were synthesized as shown in Scheme 1.

The starting 7-methoxycoumarin-3-carboxylic acid **1a** and 7-diethylaminocoumarin- 3-carboxylic acid **1b** were prepared according to the literature procedures by the Knoevenagel condensation reaction of 4-substituted salicylaldehyde with diethyl malonate [93], or with ethyl cyanoacetate [94], respectively, followed by hydrolysis reactions. Coumarins **1a,b** undergo amidation reaction with 2-azidoethylamine hydrochloride in refluxing acetonitrile in the presence of Et_3_N as a base. The reaction progress was monitored by thin layer chromatography. It should be noted that, after the cooling of the reaction mixture, the products precipitate from the reaction mixture, and in the case of derivative **2a**, in the form of crystals suitable for single crystal X-ray diffraction study. The structures of the obtained compounds **2a,b** were established using IR, ^1^H NMR, and ^13^C NMR spectroscopy (see Supplementary Materials). The ^1^H NMR spectra of each compound in CDCl_3_ contain, in addition to characteristic signals of the coumarin moiety, broad triplets of the NH-amide protons at 9.0 ppm and signals from the two side chain methylene groups at ~3.6 and 3.5 ppm. In the IR spectra, the characteristic absorption bands of the N_3_ group appear at 2125 and 2098 cm^−1^ for **2a** and **2b**, respectively.

**Scheme 2:**
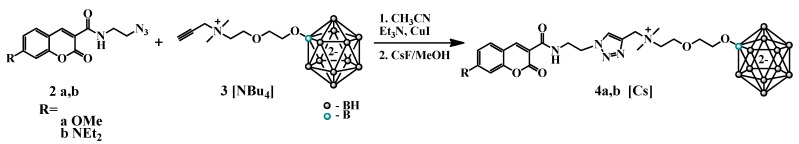
“Click” reactions of coumarins **2a** and **2b** with the alkyne *closo*-dodecaborate derivative **3**[NBu_4_].

To demonstrate the ability of novel azido coumarins **2a,b** to form conjugates with fluorescent properties, we have tested them in reactions with a *closo*-dodecaborate derivative containing a terminal alkynyl group in the side chain **3**[NBu_4_], under typical “click” reaction conditions. As a result, new 1,2,3-triazoles **4a**[Cs] and **4b**[Cs] were obtained in high yields (Scheme 2).

The formation of conjugates **4a,b** was monitored by the TLC method, and the full conversion was gained in 16 h. The conjugation products were isolated by precipitation in the form of the Cs^+^ salts and characterized by ^1^H, ^11^B, ^13^C NMR, and IR spectroscopy as well as high-resolution mass spectrometry (see Supplementary Materials). The formation of the 1,2,3-triazole heterocycle results in the appearance of the corresponding singlets at ~ 8.45 ppm in the ^1^H NMR spectra. In the IR spectra of **4a,b**, the absorption bands of the BH stretching (2487 cm^−1^) demonstrate the presence of the boron hydride cluster, and absorption bands corresponding to the 1,2,3-triazole heterocycle (1705 and 1710 cm^−1^, respectively) were detected. In contrast to azido-coumarins **2a,b**, fluorescent conjugates **4a,b** possess a good solubility in water.

### 2.3. Single Crystal X-ray Diffraction Study of Azido Derivatives of Coumarins

**Figure 2:**
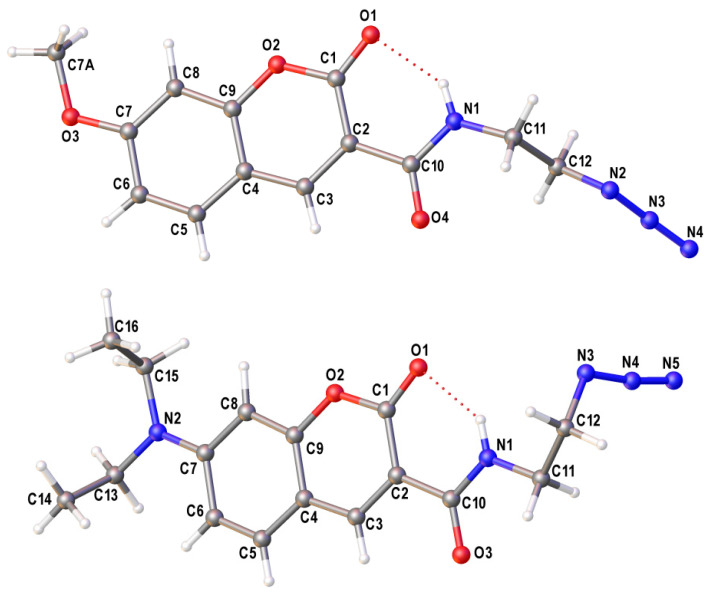
Molecular crystal structures of **2a** (**top**) and **2b** (**bottom**). Non-hydrogen atoms are presented by probability ellipsoids of atomic displacements (*p* = 0.5). Dotted lines correspond to the intramolecular hydrogen bonds.

The molecular structure of the obtained azido derivatives of coumarins **2a** and **2b** were determined by a single crystals X-ray diffraction study (Figure 2).

**Figure 3:**
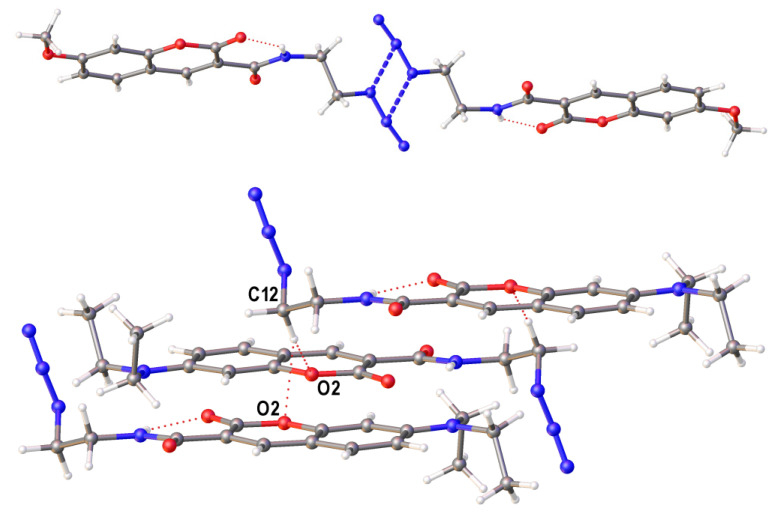
Fragments of crystal packing of **2a** (**top**) and **2b** (**bottom**) demonstrating the N_3_…N_3_ stacking interactions in **2a** (dashed bold lines) and bifurcate C12(H)…O2 H-bond in **2b** (dotted lines).

The amide fragments in both compounds are expectedly planar: the mean deviation of non-hydrogen atoms from the corresponding mean-square planes are only 0.02 and 0.04 Å for **2a** and **2b**, respectively. The most significant deviations from the coumarin-acetylamide planes are observed for the atoms of amide moiety (0.03 Å for N1 in **2a** and 0.10 Å for O3 in **2b**) that can be rationalized by the formation of intramolecular N1(H)…O1 hydrogen bonds between the amide hydrogen and the coumarin carbonyl (∠(NHO) 138.1° and 131.6°, N…O 2.751 and 2.749 Å in **2a** and **2b**, respectively), which is typical for primary 3-arylamido- and 3-hydrazidocoumarins [95,96,97,98]. The conformations of the azidoethyl fragments are pronouncedly different in **2a** and **2b**: while the *anti*-conformation is observed for the substituents at C11-C12 bond in **2a** (the N1-C11-C12-N2 torsion angle is 170.5(1)°), the *gosh*-conformation is observed in **2b** (the corresponding torsion angle is 61.2(1)°). In its turn, the N_3_ group is antiperiplanar to the C11-C12 bond in **2a** (175.0(1)°) and to the C12-H12A bond in **2b** (173.8(1)°). This difference can be readily explained by the peculiarities of crystal packing (Figure 3): in **2b**, the C12(H) group participates in CH…O contacts with O2 oxygen atoms of two neighboring molecules, whereas the only meaningful interaction for the azidoethyl fragment in **2a** is the stacking interaction between N_3_ moieties (N…N 3.07 Å), which were also observed in some other organic compounds with the azido group [99,100]. In general, the morphology of crystal packings of **2a** and **2b** are still similar (see Supplementary Materials, Figure S1): the layer-type arrangement of molecules is stabilized by stacking interactions between coumarin acylamide fragments and CH…O hydrogen bonds.

### 2.4. Photophysical Properties

**Figure 4:**
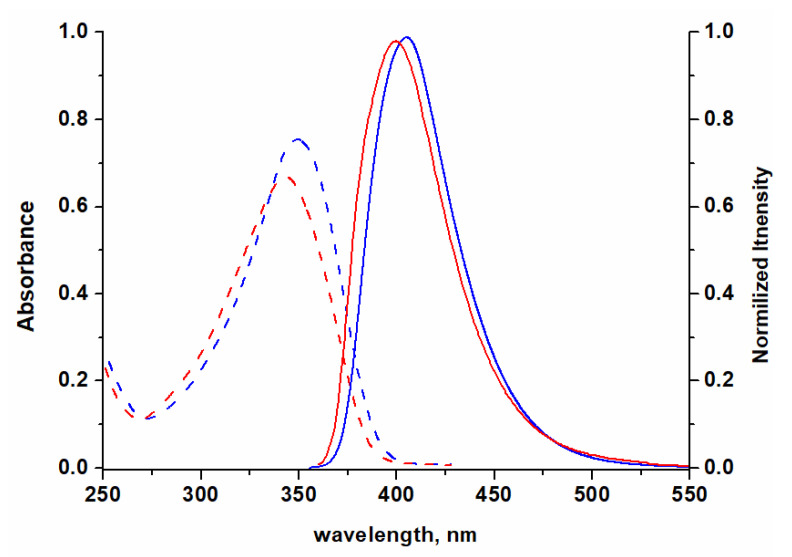
UV–vis (dashed line) and normalized emission (solid line) spectra of coumarin **2a** in CH_3_CN (c = 1.5 × 10^−4^ M, blue) and conjugate **4a** in water (c = 0.5 × 10^−4^ M, red).

**Figure 5:**
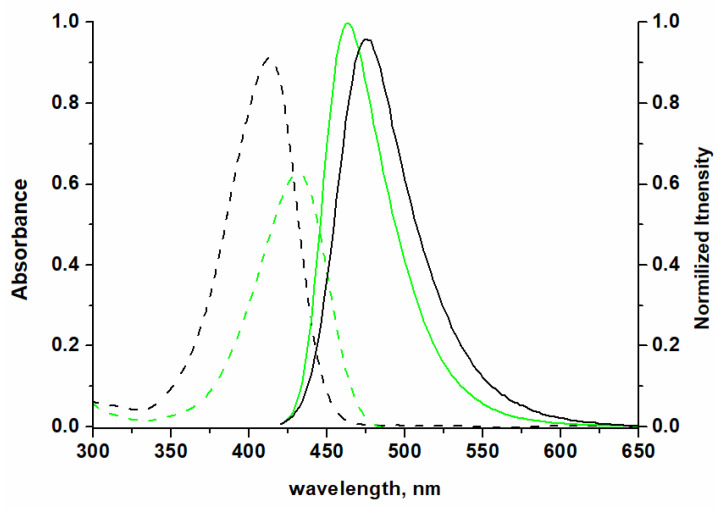
UV–vis (dashed line) and normalized emission (solid line) spectra of coumarin **2b** in CH_3_CN (c = 1 × 10^−4^ M, black) and conjugate **4b** in water (c = 0.5 × 10^−4^ M, green).

The UV–vis spectra of compounds **2a** and **2b** were measured in acetonitrile. In contrast, the derivatives **4a** and **4a** are soluble in water, and their absorption spectra were measured in water correspondingly (Figure 4). All compounds demonstrate intense bands in the short wavelength region of the π → π* character within the coumarin aromatic system. Differences at the absorption maxima suggest that the donor–acceptor properties of the 7-substituted group affect the excitation energy. Compounds **2a** and **4a** with -OMe substituent demonstrate bands at 350 and 343 nm respectively. Compounds **2b** and **4b** containing -NEt_2_ substituent demonstrate bands in the lower energy region (413 and 430 nm, respectively). Compounds **2a**, **2b** in CH_3_CN, and **4a**, **4b** in water demonstrate unstructured broad bands centered at ca. 400 and 470 nm for OMe and NEt_2_ substituted analogues, respectively (Figure 5, Table 1). The emission observed is a fluorescence, being typical for substituted coumarins [101,102]. Differences at the emission maxima demonstrate the effects of the donor properties of substituent on the emission. The 7–10 nm of the wavelength difference between **2a** and **4a** as well as between **2b** and **4b** may be explained by the solvent effect.

### 2.5. Antiproliferative Activity and Cell Uptake of Boronated Coumarins

**Figure 6:**
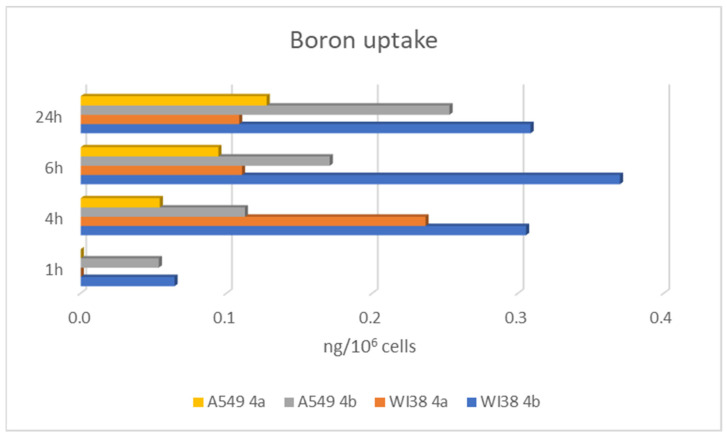
Cellular boron accumulation of compounds **4a** and **4b** in A549 and WI38 cells.

The antiproliferative activity of compounds **4a** and **4b** against the human HCT116 colorectal carcinoma, MCF7 breast adenocarcinoma, A549 non-small cell lung carcinoma and WI38 nonmalignant lung fibroblast cell lines was evaluated by means of a standard MTT colorimetric assay as the IC_50_ value after 72 h of incubation. Compounds **4a** and **4b** were found to be not active up to 200 µM (See Supplementary Materials) and can be considered as non-toxic against cancerous and non-malignant cells. Therefore, they could be of potential interest for use in BNCT for cancer. Cellular accumulation plays a fundamental role in the antiproliferative activity of low-molecular-mass compounds [103,104]. One of the main requirements of effective BNCT is the selective accumulation of boron-containing compounds in the tumor with a fairly high concentration (approx. 10^9 10^B atoms/cell or 20–35 µg/gram [60]). Therefore, we measured uptake of compounds **4a** and **4b** at 250 µM concentration in A549 and WI38 cell lines for 1, 4, 6 and 24 h; the amount of boron was determined by ICP-MS (Figure 6).

The accumulation of compound **4b** in both cancerous and normal cells occurs noticeably faster than compound **4a** and reaches a maximum after 6 h for the WI38 cell line and after 24 h for the A549 cell line. At the same time, both compounds accumulated slightly better in the WI38 cell line. The cells of many tumours are known to be much larger than normal cells [105,106]; however, the close size of the A549 cells [107] and red blood cells [108] allows a rough estimate of the accumulation of the compound **4b** in the cancer cells, which does not exceed 10 μg/g, which is apparently insufficient for effective BNCT.

## 3. Materials and Methods

Chemicals were reagent grade and received from commercial vendors. Acetonitrile was distilled from P_2_O_5_ and then from CaH_2_. Compounds **1a** [93], **1b** [94], 2-azidoethylamine hydrochloride [109] and **3** [92] were prepared according to the literature procedures. All reactions were carried out in air. Analytical TLC was performed on Kieselgel 60 F245 (Merck, Darmstadt, Germany) plates. Boron compounds were visualized with PdCl_2_ stain solution, which upon heating gave dark brown spots. ^1^H, ^13^C and ^11^B NMR spectra were recorded at 400.13, 100.61 and 128.38 MHz, respectively, on a BRUKER-Avance-400 spectrometer (Bruker, Karlsruhe-Zurich, Switzerland-Germany). Tetramethylsilane and BF_3_ × Et_2_O were used as standards for ^1^H and ^13^C NMR and ^11^B NMR, respectively. All chemical shifts are reported in ppm (δ) relative to external standards. IR spectra were recorded on IR Prestige-21 (Shimadzu, Kyoto, Japan) instrument. The UV–vis spectra of solutions were measured on Cary 50 Uv–Vis Spectrophotometer (Varian, Palo Alto, CA, USA). The photoluminescence spectra were recorded on the Spectrofluorometer RF-6000 (Shimadzu). High resolution mass spectra (HRMS) were measured on a Bruker micrOTOF II instrument (Bruker Daltonic, Bremen, Germany) using electrospray ionization (ESI). For anionic boron containing compounds, the measurements were performed in a negative ion mode (3200 V) with a mass range from *m*/*z* 50 to *m*/*z* 3000; external and internal calibration was performed with ESI Tuning Mix (Agilent, Santa Clara, CA, USA). The boron content in cells was determined by inductively coupled plasma mass spectrometry (ICP-MS) using X-II spectrometer (Thermo Scientific, Waltham, MA, USA) and the following parameters: RF generator power 1400 W, nebulizer PolyCon, spray chamber cooling 3 °C, plasma gas flow rate 12 L/min, auxiliary flow rate 0.9 L/min, nebulizer flow rate 0.9 L/min, sample update 0.8 mL/min, resolution 0.8 M. The main parameters of MS measurements were: detector mode double (pulse counting and analogous) and scanning mode Survey Scan and Peak Jumping. The settings for the Survey Scan were: number of runs 10, dwell time 0.6 ms, channels per mass 10, acquisition 13.2 s. The settings for the Peak Jumping were: sweeps 25, dwell time 10 ms, channels per mass 1, acquisition 34 s.

### 3.1. Synthetic Procedures

#### 3.1.1. *N*-(2-azidoethyl)-7-methoxy-2-oxo-2H-chromene-3-carboxamide **2a**

To a suspension of 3-carboxy-7-methoxycoumarin **1a** (0.5 g, 2.3 mmol) and 2-azidoethanamine hydrochloride (0.31 g, 2.5 mmol) in 50 mL of acetonitrile, NEt_3_ was added in excess (approximately 0.5 mL). After the amine addition, the solution became clear. The reaction mixture was stirred under reflux for 16 h. Pale yellow crystals were formed after a slow cooling to room temperature; they were filtered washed with water (5 mL), cold CH_3_CN (5 mL) and air dried to give 0.32 g of **2**. Another portion of **2a** was purchased from the mother liquor by recrystallisation from EtOH to give while combining 0.40 g of **2a**. Colorless solid. Yield: 61%.

^1^H NMR (Chloroform-*d*, δ, ppm): δ 9.03 (s, 1H, NH), 8.85 (s, 1H, H-4), 7.61 (d, *J* = 8.7 Hz, 1H, H-5), 6.67 (dd, *J* = 8.7, 2.4 Hz, 1H, H-6), 6.88 (d, *J* = 2.5 Hz, 1H, H-8), 3.93 (s, 3H, OMe), 3.66 (q, *J* = 5.8 Hz, 2H, NHC*H*_2_CH_2_), 3.56 (t, *J* = 5.8 Hz, 2H, NHCH_2_C*H*_2_). ^13^C NMR (Chloroform-*d*, δ, ppm): 165.0, 162.5 (CO), 161.8 (C*), 156.7 (C-7), 148.5 (C-4), 131.0 (C-5), 114.3 (C-6), 114.1 (C*), 112.3 (C-3), 100.3 (C-8), 56.1 (OCH_3_), 50.6 (N*C*H_2_CH_2_), 39.1 (NCH_2_*C*H_2_). IR (KBr, ν, cm^−1^): δ 3353 (NH), 3086, 3050, 2986, 2946 (CH), 2125 (N3), 1713, 1658 (CO), 1609 (C=C).

#### 3.1.2. *N*-(2-azidoethyl)-7-(diethylamino)-2-oxo-2H-chromene-3-carboxamide **2b**

To a suspension of 3-carboxy-7-diethylaminocoumarin **1b** (0.5 g, 1.9 mmol) and 2-azidoethanamine hydrochloride (0.26 g, 2.1 mmol) in 50 mL of acetonitrile, NEt_3_ was added in excess (approximately 0.5 mL). After the amine addition, the solution became clear. The reaction mixture was stirred under reflux for 16 h. Then it was allowed to cool to room temperature. Yellow precipitate was formed after a cooling of the reaction mixture to 0 °C; it was filtered washed with water (5 mL), cold CH_3_CN (5 mL) and air dried. Mother liquor was evaporated to dryness and the oily residue was recrystallized from the EtOAc-EtOH mixture to give an additional portion of the title product. Orange solid. Yield: 63%. 0.4 g Crystals were obtained from the EtOAc solution while in slow evaporation.

^1^H NMR (Chloroform-*d*, δ, ppm): δ 9.06 (s, 1H, NH), 8.72 (s, 1H, H-4), 7.45 (d, *J* = 9.0 Hz, 1H, H-5), 6.67 (dd, *J* = 8.9, 2.5 Hz, 1H, H-6), 6.52 (d, *J* = 2.5 Hz, 1H, H-8), 3.65 (q, *J* = 5.8 Hz, 2H, NHC*H*_2_CH_2_), 3.55 (t, *J* = 5.9 Hz, 2H, NHCH_2_C*H*_2_), 3.48 (q, *J* = 7.1 Hz, 4H, NC*H*_2_CH_3_), 1.26 (t, *J* = 7.1 Hz, 6H, NCH_2_C*H*_3_). ^13^C NMR (Chloroform-*d*, δ, ppm): 163.6, 162.7 (CO), 157.7 (C*), 152.7 (C-7), 148.3 (C-4), 131.2 (C-5), 110.0 (C-6), 109.8 (C*), 108.3 (C-3), 96.6 (C-8), 50.7 (N*C*H_2_CH_2_), 45.1 (*C*H_2_CH_3_), 39.0 (NCH_2_*C*H_3_), 12.4 (CH_2_*C*H_3_). IR (KBr, ν, cm^−1^): δ 3357 (NH), 2986, 2942, 2883(CH), 2097 (N3), 1697, 1649 (CO), 1626 (C=C), 1590, 1525.

#### 3.1.3. Synthesis of Conjugate **4a**

A mixture of alkyne **3** (NBu_4_) (0.1 g, 0.2 mmol), **2a** (0.052 g, 0.2 mmol), Et_3_N (0.5 mL) and CuI (0.01 g, 0.04 mmol) in 15 mL of CH_3_CN was stirred under reflux for 16 h. The reaction mixture was allowed to cool to room temperature and passed through a layer of silica gel (2–3 cm) on a Schott filter. The system was washed with CH_3_CN until the product ceased to be detected by thin layer chromatography. The solvent was removed on a rotary evaporator. The residue was dissolved in MeOH (10 mL) and an excess of CsF solution in MeOH was added. Formed precipitate was filtered, washed with MeOH (20 mL) and air dried to yield title **4a** as a pale-yellow solid. 0.12 g. Yield: 91%.

^1^H NMR (DMSO-*d*_6,_ δ, ppm): 8.83 (s, 1H, NH), 8.73 (s, 1H, H-4), 8.46 (s, 1H, CH-triazole), 7.93 (d, *J* = 8.7 Hz, 1H, H-5), 7.13 (d, *J* = 2.5 Hz, 1H, H-8), 7.05 (dd, *J* = 8.9 Hz, 1H, H-6), 4.71 (s, 2H, N^+^CH_2_), 4.63 (m, 2H, N^+^CH_2_-spacer), 3.91 (m, 5H, BOCH_2_ + OCH_3_), 3.81 (m, 2H, CONC*H*_2_CH_2_), 3.46 (s, 6H, 2×CH_2_-spacer + CONCH_2_C*H*_2_), 3.10 (s, 6H, N^+^CH_3_). ^11^B NMR (DMSO-*d*_6_, δ, ppm): 6.3 (s,1B, B(1)); −16.8 (d, *J* = 149 Hz, 5B, B(2-6)); −18.1 (d, *J* = 158 Hz, 5B, B(7-11)); −22.7 (d, J = not resolved, 1B, B(12)). ^13^C NMR (DMSO-*d*_6,_ δ, ppm): 165.0, 162.3 (CO), 161.0 (C*), 156.7 (C-7), 148.4 (C-4), 136.0 (C-triazole), 132.2 (CH-triazole), 129.5 (C-5), 114.9 (C-6), 114.2 (C*), 112.5 (C-3), 100.8 (C-8), 72.3, 68.3, 64.7 (OCH_2_), 62.8, 58.8 (N^+^CH_2_), 56.8 (N*C*H_2_CH_2_), 51.1 (N^+^CH_3_), 49.9 (NCH_2_*C*H_2_). IR (KBr, ν, cm^−1^): 3437 (NH), 2942, 2867 (CH), 2487 (BH), 1705 (triazole), 1617 (CO). ESI-MS, *m*/*z*, C_22_H_40_B_12_N_5_O_6_ calcd. 600.4185 [M]^−^, found 600.4185 [M]^−^.

#### 3.1.4. Synthesis of Conjugates **4b**

A mixture of alkyne **3** (NBu_4_) (0.1 g, 0.2 mmol), **2b** (0.06 g, 0.2 mmol), Et_3_N (0.5 mL) and CuI (0.01 g, 0.04 mmol) in 15 mL of CH_3_CN was stirred under reflux for 16 h. Then it was allowed to cool to room temperature and passed through a layer of silica gel (2–3 cm) on a Schott filter. The system was washed with CH_3_CN until the product ceased to be detected by thin layer chromatography. The solvent was removed on a rotary evaporator. The residue was dissolved in MeOH (10 mL) and an excess of CsF solution in MeOH was added. Formed precipitate was filtered (attention! very fine powder), washed with MeOH (20 mL) and air dried to yield title **4b** as yellow solid. 0.09 g. Yield: 63%.

^1^H NMR (DMSO-*d*_6,_ δ, ppm): δ 8.78 (t, *J* = 5.7 Hz, 1H, NH), 8.54 (s, 1H, H-4), 8.44 (s, 1H, CH-triazole), 7.69 (d, *J* = 9.0 Hz, 1H, H-5), 6.80 (m, 1H, H-6), 6.62 (d, *J* = 2.4 Hz, 1H, H-8), 4.71 (s, 2H, N^+^CH_2_), 4.62 (t, *J* = 5.7 Hz, 2H, N^+^CH_2_-spacer), 3.92 (m, BOCH_2_), 3.80 (m, 2H, CONC*H*_2_CH_2_), 3.47 (m, 10H, 2×CH_2_-spacer, CONCH_2_C*H*_2,_ NC*H*_2_CH3), 3.10 (s, 6H, N^+^CH_3_), 1.15 (t, *J* = 7.0 Hz, 6H, NCH_2_C*H*3), 1.7–0.5 (broad m. 11H, BH). ^11^B NMR (DMSO-*d*_6_, δ, ppm): 6.4 (s,1B, B(1)); −16.8 (d, *J* = 151 Hz, 5B, B(2-6)); −18.1 (d, *J* = 152 Hz, 5B, B(7-11)); −22.7 (d, J = not resolved, 1B, B(12)). ^13^C NMR (DMSO-*d*_6,_ δ, ppm): 163.1, 161.9 (CO), 157.7 (C*), 153.0 (C-7), 148.2 (C-4), 136.0 (C-triazole), 132.2 (CH-triazole), 129.4 (C-5), 110.7 (C-6), 109.3 (C*), 108.0 (C-3), 96.3 (C-8), 72.3, 68.3, 64.7 (OCH_2_), 62.8, 58.86 (N^+^CH_2_), 51.1 (N^+^CH_3_), 50.1 (NCH_2_*C*H_2_), 44.8 (N*C*H_2_CH_3_), 12.8 (NCH_2_*C*H_3_). IR (KBr, ν, cm^−1^): 3337 (NH), 2978, 2939, 2874 (CH), 2487 (BH), 1710 (triazole). ESI-MS, *m/z*, C_25_H_47_B_12_N_6_O_5_ calcd. 641.4815 [M]^−^, found 641.4812 [M]^−^.

### 3.2. In Vitro Antiproliferative Assays and Cellular Uptake

The human HCT116 colorectal carcinoma, MCF7 breast adenocarcinoma, A549 non-small cell lung carcinoma, and WI38 nonmalignant lung fibroblast cell lines were obtained from the European collection of authenticated cell cultures (ECACC; Salisbury, UK). All cells were grown in DMEM medium (Gibco™, Irland) supplemented with 10% fetal bovine serum (Gibco™, Brazil). The cells were cultured in an incubator at 37 °C in a humidified 5% CO_2_ atmosphere and subcultured 2 times a week. The effect of the investigated compounds on cell proliferation was evaluated using a common MTT assay. The cells were seeded in 96-well tissue culture plates («TPP», Switzerland) at a 1 × 10^4^ cells/well in 100 µL of the medium. After overnight incubation at 37 °C, the cells were treated with the tested compounds in the concentration range of 0 to 200 µM. Cisplatin was used as a standard. After 72 h of treatment, solution was removed, a freshly diluted MTT solution (100 µL, 0.5 mg/mL in cell medium) was added to the wells, and the plates were further incubated for 50 min. Subsequently, the medium was removed, and the formazan product was dissolved in 100 μL of DMSO. The number of living cells in each well was evaluated by measuring the absorbance at 570 nm using the «Zenith 200 rt» microplate reader (Biochrom, Cambridge, UK). The cellular accumulation of the studied compounds was determined as described earlier [110]. Briefly, A549 and WI38 cells were seeded in 25 cm^2^ cell flask (1 × 10^6^ cells per flask). After 48 pre-incubations, the cells were exposed to compounds for 1, 4, 6, and 24 h. After the treatment, the cells were detached by trypsinization, exhaustively washed with ice-cold PBS (10mM, pH 7.4), counted by using cell counter, and collected by centrifugation. Finally, the cell pellets were digested, and the quantity of boron taken up by the cells was determined by ICP-MS.

### 3.3. Crystallography

X-ray diffraction data for crystals of **2a** and **2b** were measured using the Bruker APEX II diffractometer (Bruker AXC, Maddison, WI, USA) equipped with the Photon 2 detector (MoKα-radiation, graphite monochromator, ω-scans). The intensity data were integrated by the SAINT program [111] and were corrected for absorption and decay using SADABS [112]. Both structures were solved by direct methods using SHELXS [113] and refined using the full matrix least squares technique against F^2^ using SHELXL-2018. Positions of hydrogen atoms were found from the difference Fourier synthesis of electron density. Non-hydrogen and hydrogen atoms were refined in the anisotropic and isotropic approximations, respectively. The main refinement details and parameters are given in the Table S1 in Supporting Information. **CCDC 2173129-2173130** contain all additional supplementary data.

## 4. Conclusions

We have reported synthesis, X-ray structure study and photophysical properties of novel coumarins functionalized with azido group which are suitable for the fluorescent labeling of compounds containing terminal alkynyl group. Their representative application was performed on the “click” reaction with the *closo*-dodecaborate based alkynyl derivative. The antiproliferative activity and uptake of the synthesized boron compounds against several human cell lines were evaluated. Both compounds were found to be non-toxic against cancerous and non-malignant cells, however their cellar uptake is not sufficient for effective BNCT treatment.

## Figures and Tables

**Table 1:** Photophysical parameters for compounds **2a**, **2b**, **4a** and **4b**.

	λ_max_^abs^ (ε∙10^−3^, M^−1^ cm^−1^), nm	λ_max_^em^ (λ^ex^), nm
**2a**	350 (5.0)	398 (340)
**4a**	343 (13.4)	405 (340)
**2b**	413 (9.1)	464 (410)
**4b**	430 (12.6)	474 (410)

## Data Availability

The data presented in this study are available in Supplementary Materials.

## Supplementary Materials

The following supporting information can be downloaded at: https://www.mdpi.com/article/10.3390/molecules27238575/s1. ^1^H, ^11^B{^1^H}, ^11^B, ^13^C NMR and IR spectra of compounds **2a,b** and **4a,b**; ESI-HRMS spectra of compounds **4a,b**; Table S1 with main crystallographic data for **2a** and **2b**; Table S2 with IC_50_ values for compounds **4a,b** and cisplatin; Figure S1 with crystal packing of **2a,b**.

## Sample Availability

Samples of the compounds are available from the authors.
