# Supplementary material for: New Azido Coumarins as Potential Agents for Fluorescent Labeling and Their “Click” Chemistry Reactions for the Conjugation with closo-Dodecaborate Anion
Source: Molecules. 2022 Dec 5;27(23):8575. doi: 10.3390/molecules27238575 (PMC9738631; doi:10.3390/molecules27238575)

checkCIF/PLATON report

Structure factors have been supplied for datablock(s) a

THIS REPORT IS FOR GUIDANCE ONLY. IF USED AS PART OF A REVIEW PROCEDURE FOR PUBLICATION, IT SHOULD NOT REPLACE THE EXPERTISE OF AN EXPERIENCED CRYSTALLOGRAPHIC REFEREE.

No syntax errors found. CIF dictionary Interpreting this report

Datablock: a

```
Bond precision:   C-C = 0.0020 A          Wavelength=0.71073

Cell:           a=7.1649(3)       b=7.5821(4)       c=12.4106(6)
                alpha=95.318(2)   beta=106.326(2)   gamma=99.061(2)
Temperature:    140 K

                 Calculated                   Reported
Volume           632.24(5)                    632.24(5)
Space group      P -1                         P -1
Hall group       -P 1                         -P 1
Moiety formula   C13 H12 N4 O4                C13 H12 N4 O4
Sum formula      C13 H12 N4 O4                C13 H12 N4 O4
Mr               288.27                       288.27
Dx,g cm-3        1.514                        1.514
Z                2                            2
Mu (mm-1)        0.116                        0.116
F000             300.0                        300.0
F000'            300.15
h,k,lmax         10,10,17                     10,10,17
Nref             3682                         3669
Tmin,Tmax        0.979,0.987                  0.962,0.992
Tmin'            0.973

Correction method= # Reported T Limits: Tmin=0.962 Tmax=0.992
AbsCorr = MULTI-SCAN

Data completeness= 0.996           Theta(max)= 29.998

                                                     wR2(reflections)=
R(reflections)= 0.0455( 2091)                        0.1304( 3669)

S = 0.894                  Npar= 238
```

The following ALERTS were generated. Each ALERT has the format
test-name_ALERT_alert-type_alert-level.
Click on the hyperlinks for more details of the test.

Alert level C

```
PLAT230_ALERT_2_C Hirshfeld Test Diff for    N2       --N3       .        6.7 s.u.
PLAT905_ALERT_3_C Negative K value in the Analysis of Variance ...     -6.409 Report
```

Alert level G

```
PLAT154_ALERT_1_G The s.u.'s on the Cell Angles are Equal ..(Note)      0.002 Degree
PLAT883_ALERT_1_G No Info/Value for _atom_sites_solution_primary .     Please Do !
PLAT910_ALERT_3_G Missing # of FCF Reflection(s) Below Theta(Min).          1 Note
PLAT912_ALERT_4_G Missing # of FCF Reflections Above STh/L=  0.600         13 Note
PLAT933_ALERT_2_G Number of HKL-OMIT Records in Embedded .res File          1 Note
PLAT941_ALERT_3_G Average HKL Measurement Multiplicity ..........         3.0 Low
PLAT965_ALERT_2_G The SHELXL WEIGHT Optimisation has not Converged     Please Check
PLAT967_ALERT_5_G Note: Two-Theta Cutoff Value in Embedded .res ..       60.0 Degree
PLAT978_ALERT_2_G Number C-C Bonds with Positive Residual Density.          5 Info
```

0 **ALERT level A** = Most likely a serious problem - resolve or explain
0 **ALERT level B** = A potentially serious problem, consider carefully
2 **ALERT level C** = Check. Ensure it is not caused by an omission or oversight
9 **ALERT level G** = General information/check it is not something unexpected

2 ALERT type 1 CIF construction/syntax error, inconsistent or missing data
4 ALERT type 2 Indicator that the structure model may be wrong or deficient
3 ALERT type 3 Indicator that the structure quality may be low
1 ALERT type 4 Improvement, methodology, query or suggestion
1 ALERT type 5 Informative message, check

It is advisable to attempt to resolve as many as possible of the alerts in all categories. Often the minor alerts point to easily fixed oversights, errors and omissions in your CIF or refinement strategy, so attention to these fine details can be worthwhile. In order to resolve some of the more serious problems it may be necessary to carry out additional measurements or structure refinements. However, the purpose of your study may justify the reported deviations and the more serious of these should normally be commented upon in the discussion or experimental section of a paper or in the "special_details" fields of the CIF. checkCIF was carefully designed to identify outliers and unusual parameters, but every test has its limitations and alerts that are not important in a particular case may appear. Conversely, the absence of alerts does not guarantee there are no aspects of the results needing attention. It is up to the individual to critically assess their own results and, if necessary, seek expert advice.

Publication of your CIF in IUCr journals

A basic structural check has been run on your CIF. These basic checks will be run on all CIFs submitted for publication in IUCr journals (*Acta Crystallographica*, *Journal of Applied Crystallography*, *Journal of Synchrotron Radiation*); however, if you intend to submit to *Acta Crystallographica Section C* or *E* or *IUCrData*, you should make sure that full publication checks are run on the final version of your CIF prior to submission.

Publication of your CIF in other journals

Please refer to the *Notes for Authors* of the relevant journal for any special instructions relating to CIF submission.

PLATON version of 28/11/2022; check.def file version of 28/11/2022

Datablock a - ellipsoid plot

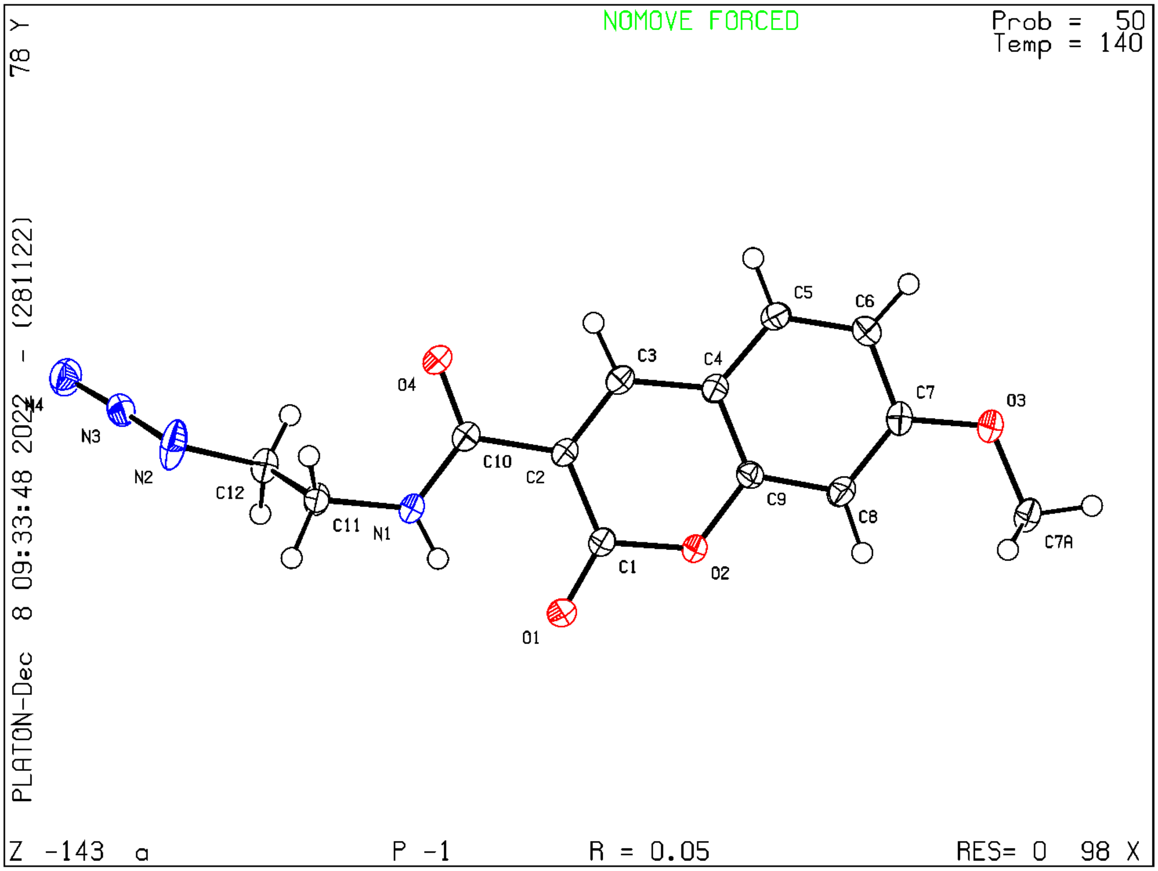

Supplement: Supplementary file 1 [file molecules-27-08575-s001.zip › checkcif_2a.pdf]
