# Supplementary material for: New Azido Coumarins as Potential Agents for Fluorescent Labeling and Their “Click” Chemistry Reactions for the Conjugation with closo-Dodecaborate Anion
Source: Molecules. 2022 Dec 5;27(23):8575. doi: 10.3390/molecules27238575 (PMC9738631; doi:10.3390/molecules27238575)
Supplement: Supplementary file 1 [file molecules-27-08575-s001.zip › Supplementary Materials.pdf]

Supporting information

New Azido Coumarins as Potential Agents for Fluorescent Labeling and Their "Click" Chemistry Reactions for the Conjugation with *closo*-Dodecaborate Anion

Julia Laskova [1,*], Alexander Serdyukov [1,2], Irina Kosenko [1], Ivan Ananyev [3], Ekaterina Titova [1], Anna Druzina [1], Igor Sivaev [1,4], Anastasia A. Antonets [5], Alexey A. Nazarov [5] and Vladimir I. Bregadze [1]

[1] A. N. Nesmeyanov Institute of Organoelement Compounds, Russian Academy of Sciences, 28 Vavilov Str., 119334 Moscow, Russia
[2] M.V. Lomonosov Institute of Fine Chemical Technology, MIREA—Technological University, 86 Vernadsky Avenue, 119571 Moscow, Russia
[3] N. S. Kurnakov Institute of General and Inorganic Chemistry, Russian Academy of Sciences, 31 Leninsky Avenue, 119991 Moscow, Russia
[4] Basic Department of Chemistry of Innovative Materials and Technologies, G. V. Plekhanov Russian University of Economics, 36 Stremyannyi Line, 117997 Moscow, Russia
[5] Department of Chemistry, M. V. Lomonosov Moscow State University, Leninskie Gory 1/3, 119991 Moscow, Russia
* Correspondence: laskova@ineos.ac.ru; Tel.: +41-78-243-1408

***N*-(2-azidoethyl)-7-methoxy-2-oxo-2H-chromene-3-carboxamide *2a*.**

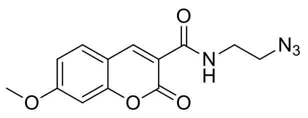

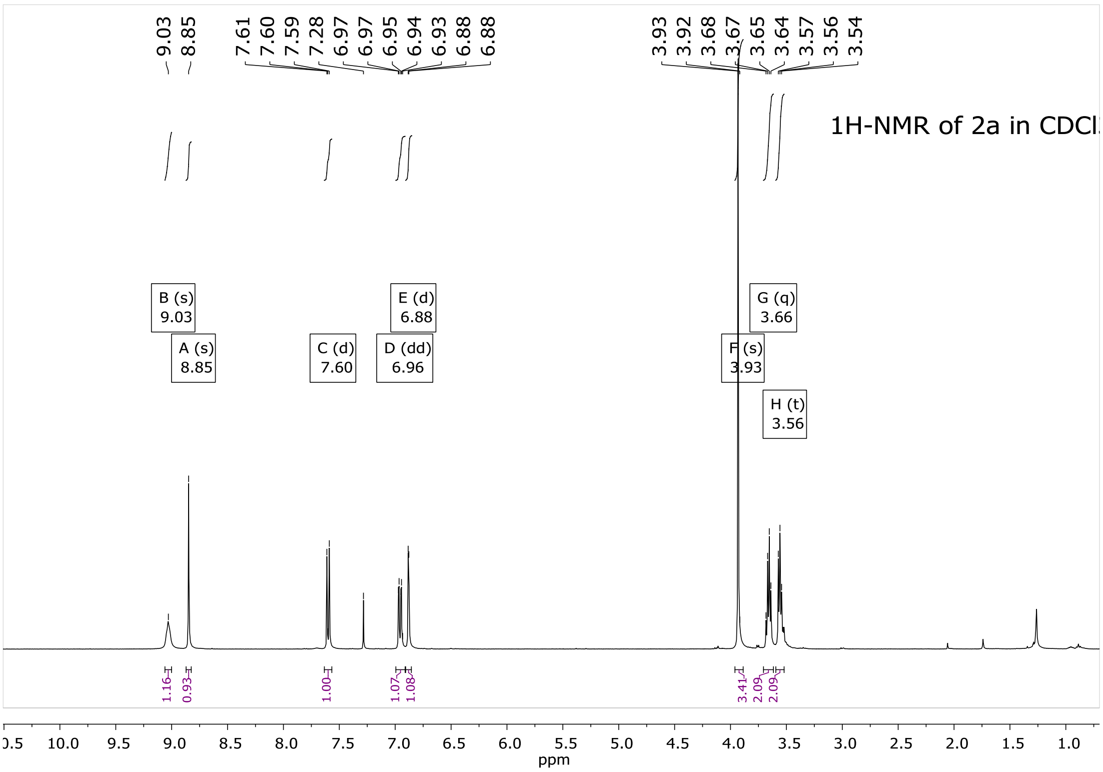

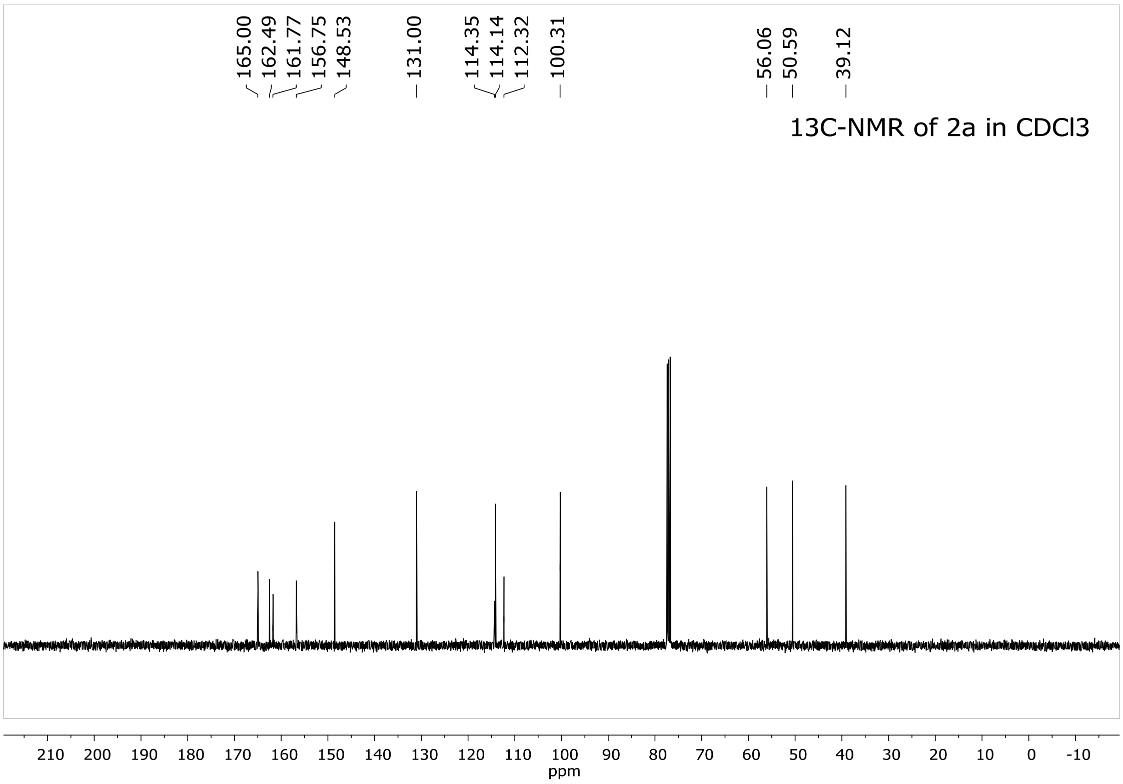

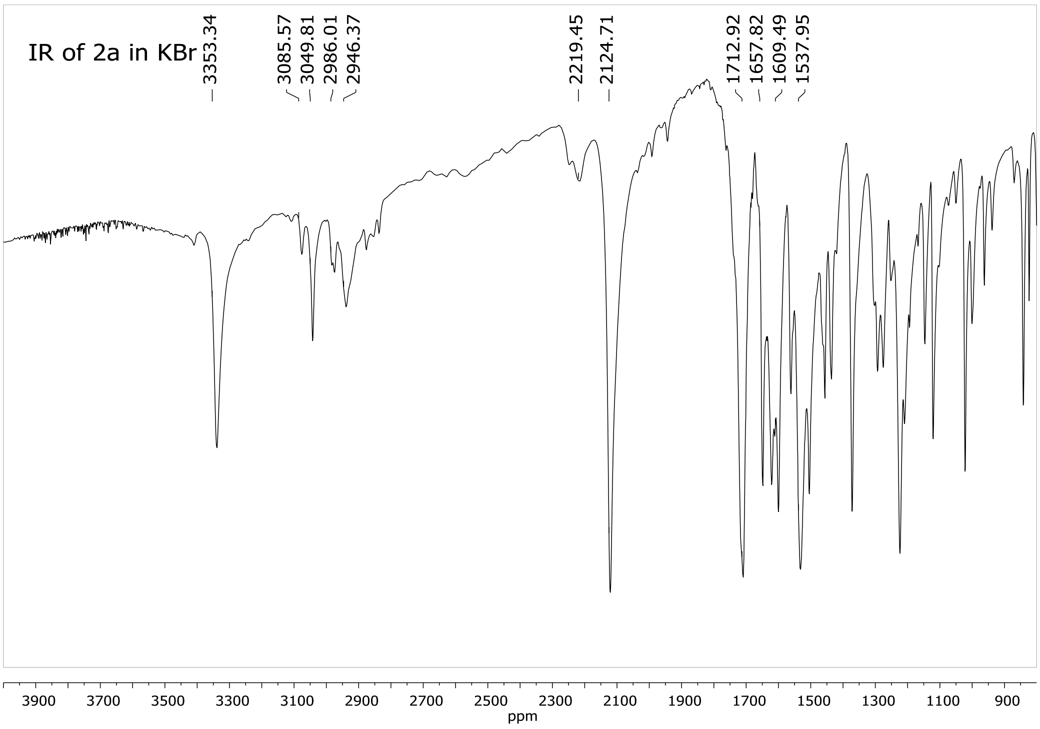

***N*-(2-azidoethyl)-7-(diethylamino)-2-oxo-2H-chromene-3-carboxamide 2b**.

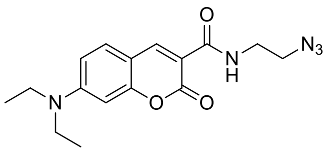

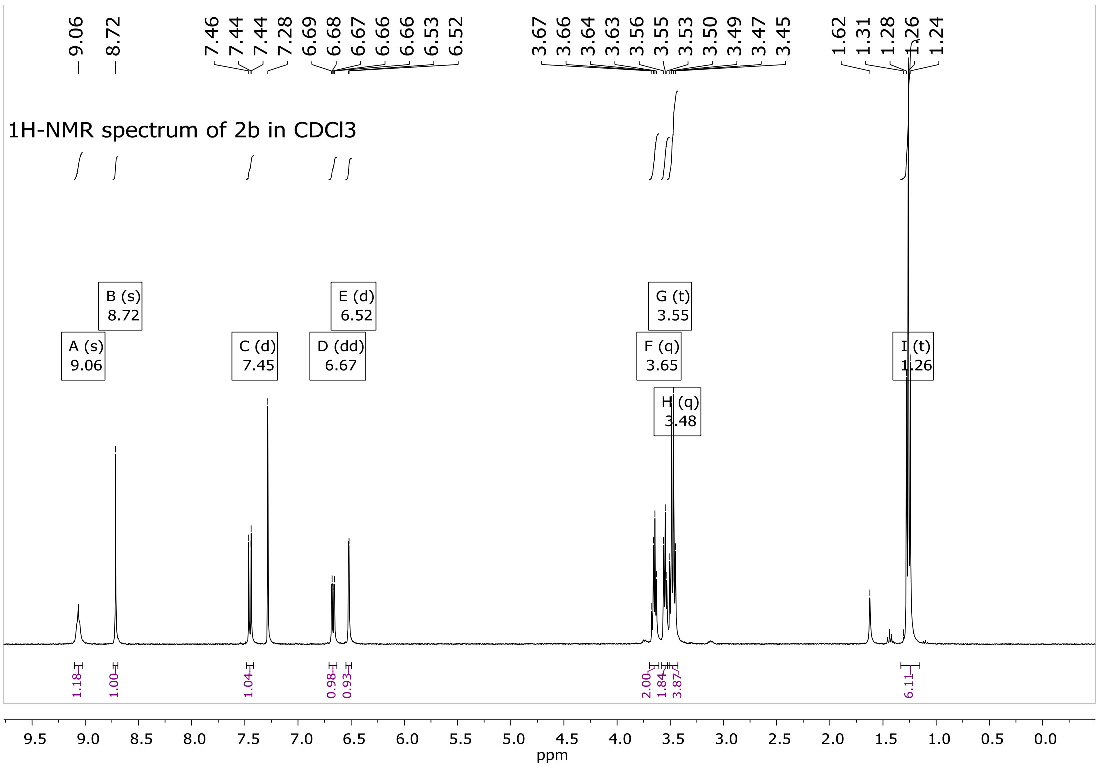

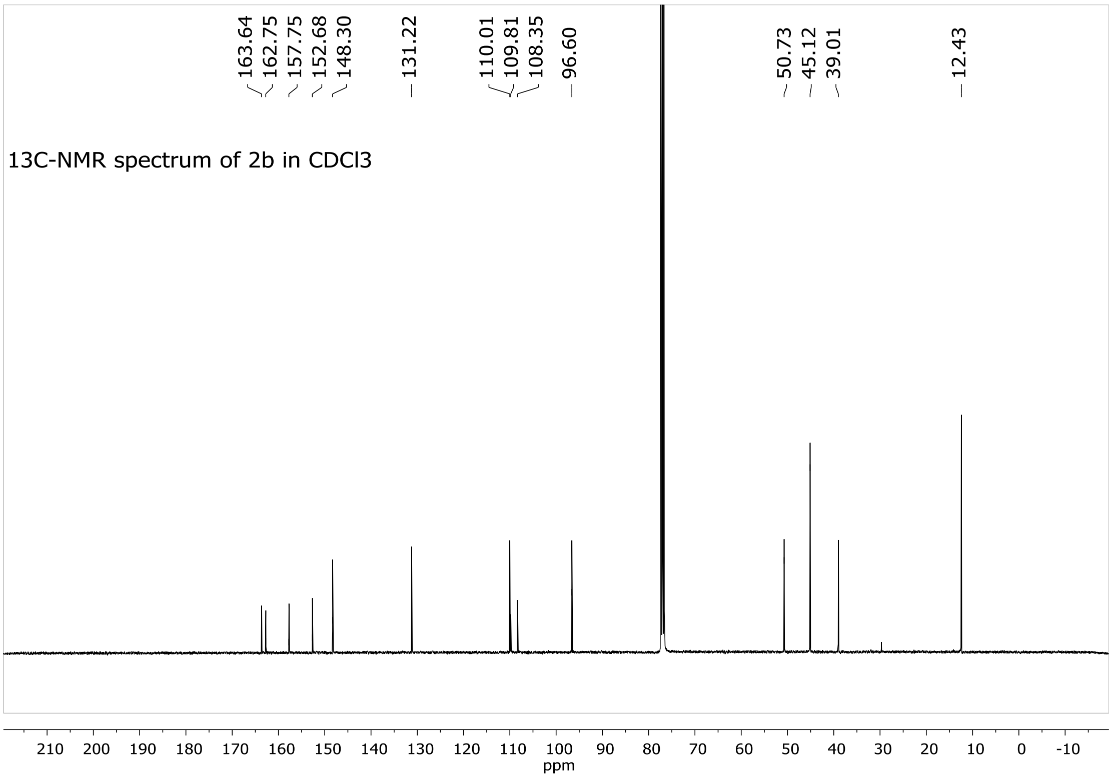
163.64
162.75
157.75
152.68
148.30
131.22
110.01
109.81
108.35
96.60
50.73
45.12
39.01
12.43
13C-NMR spectrum of 2b in CDCl3
210 200 190 180 170 160 150 140 130 120 110 100 90 80 70 60 50 40 30 20 10 0 -10
ppm

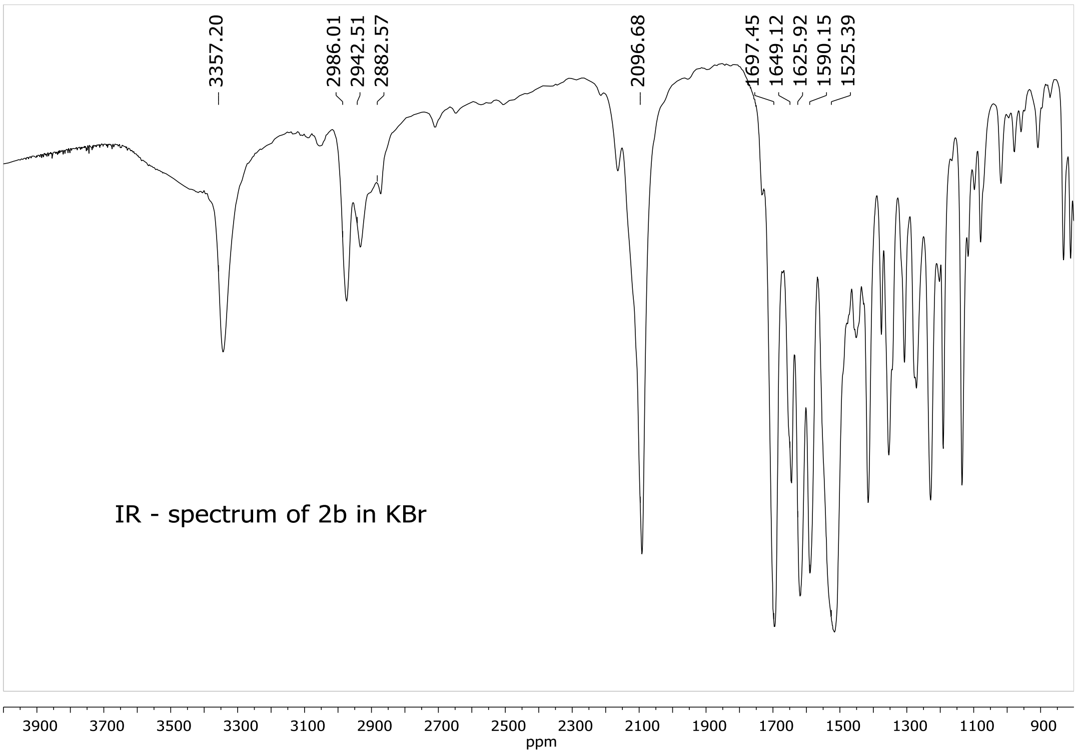
3357.20
2986.01
2942.51
2882.57
2096.68
1697.45
1649.12
1625.92
1590.15
1525.39
IR - spectrum of 2b in KBr
3900 3700 3500 3300 3100 2900 2700 2500 2300 2100 1900 1700 1500 1300 1100 900
ppm

Conjugate *4* a

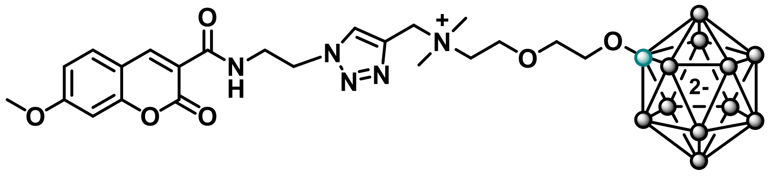

[4a]Cs

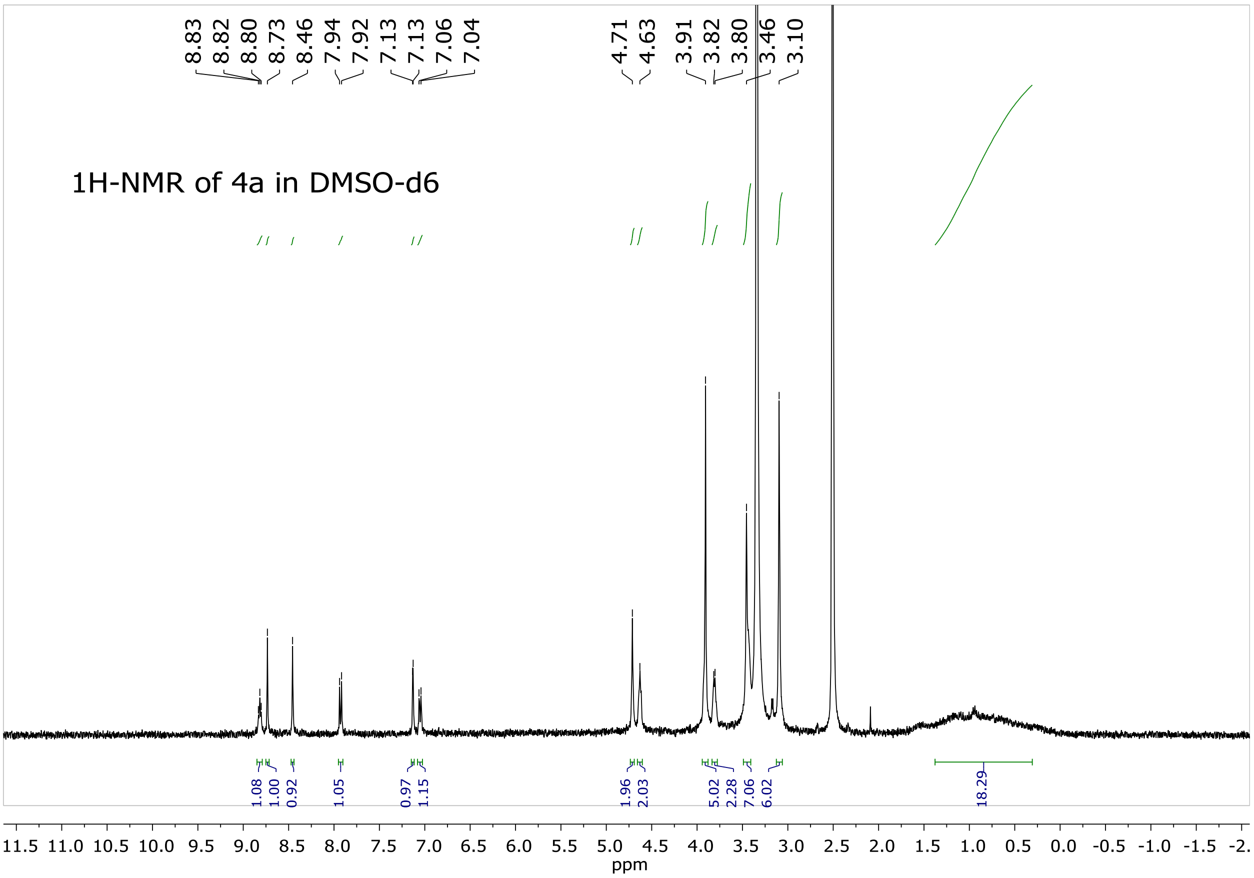

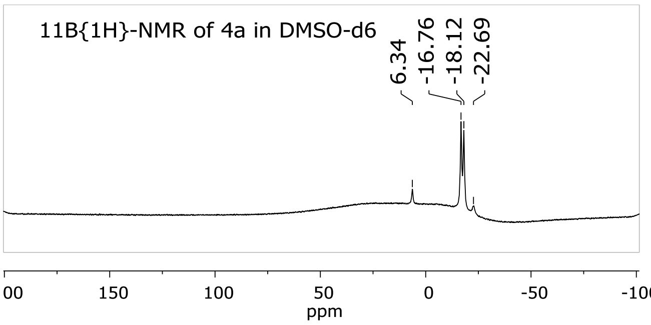

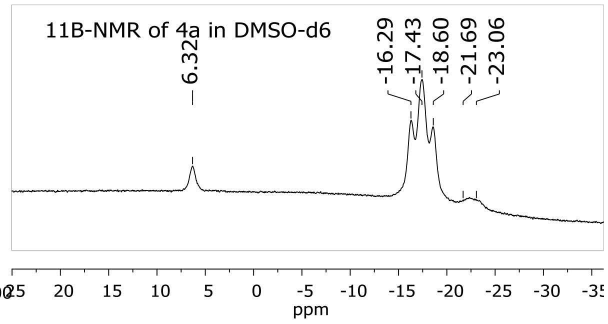

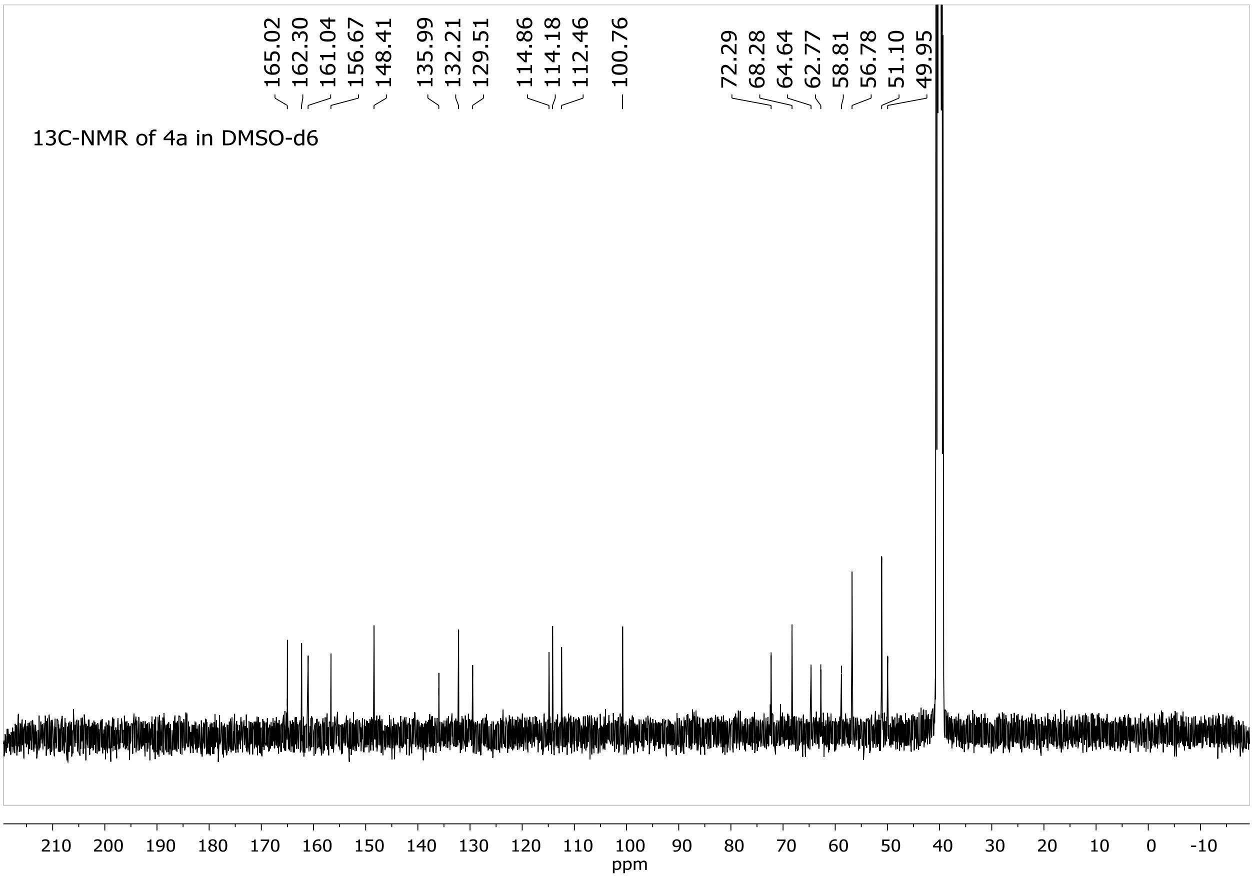

165.02
162.30
161.04
156.67
148.41
135.99
132.21
129.51
114.86
114.18
112.46
100.76
72.29
68.28
64.64
62.77
58.81
56.78
51.10
49.95
13C-NMR of 4a in DMSO-d6
210 200 190 180 170 160 150 140 130 120 110 100 90 80 70 60 50 40 30 20 10 0 -10
ppm

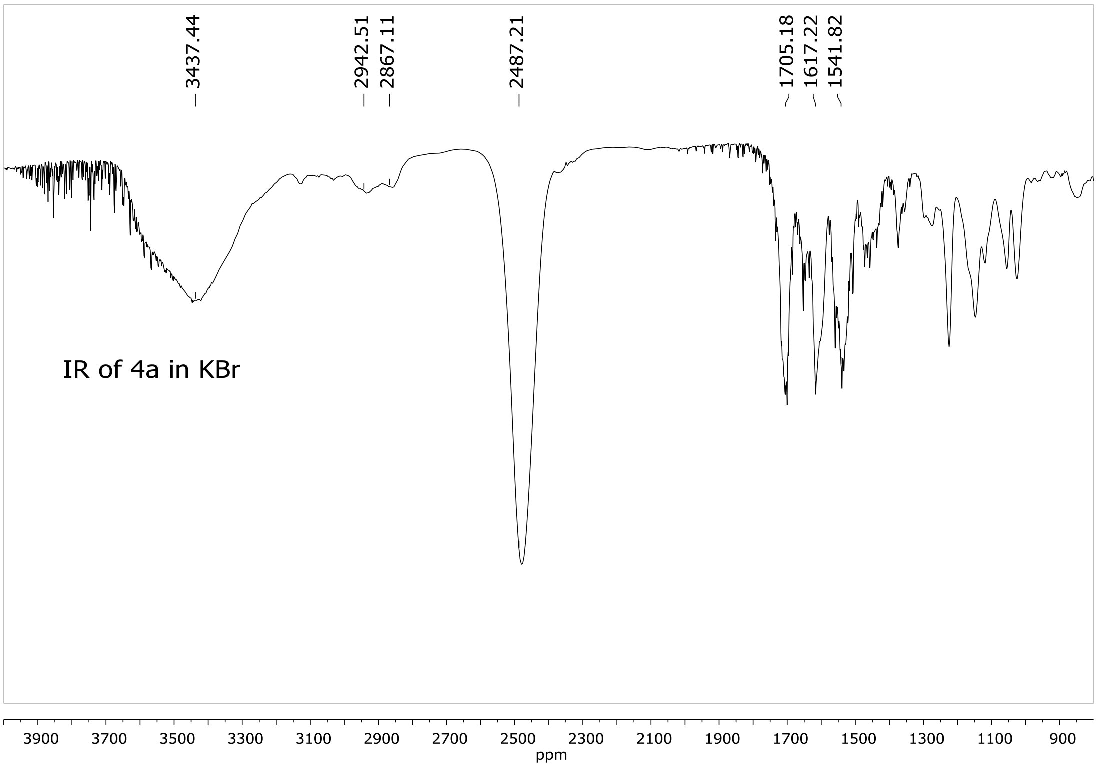

3437.44
2942.51
2867.11
2487.21
1705.18
1617.22
1541.82
IR of 4a in KBr
3900 3700 3500 3300 3100 2900 2700 2500 2300 2100 1900 1700 1500 1300 1100 900
ppm

Acquisition Parameter

Source Type	ESI	Ion Polarity	Negative	Set Nebulizer	0.4 Bar
Focus	Active			Set Dry Heater	180 °C
Scan Begin	50 m/z	Set Capillary	3000 V	Set Dry Gas	4.0 l/min
Scan End	3000 m/z	Set End Plate Offset	-500 V	Set Divert Valve	Waste

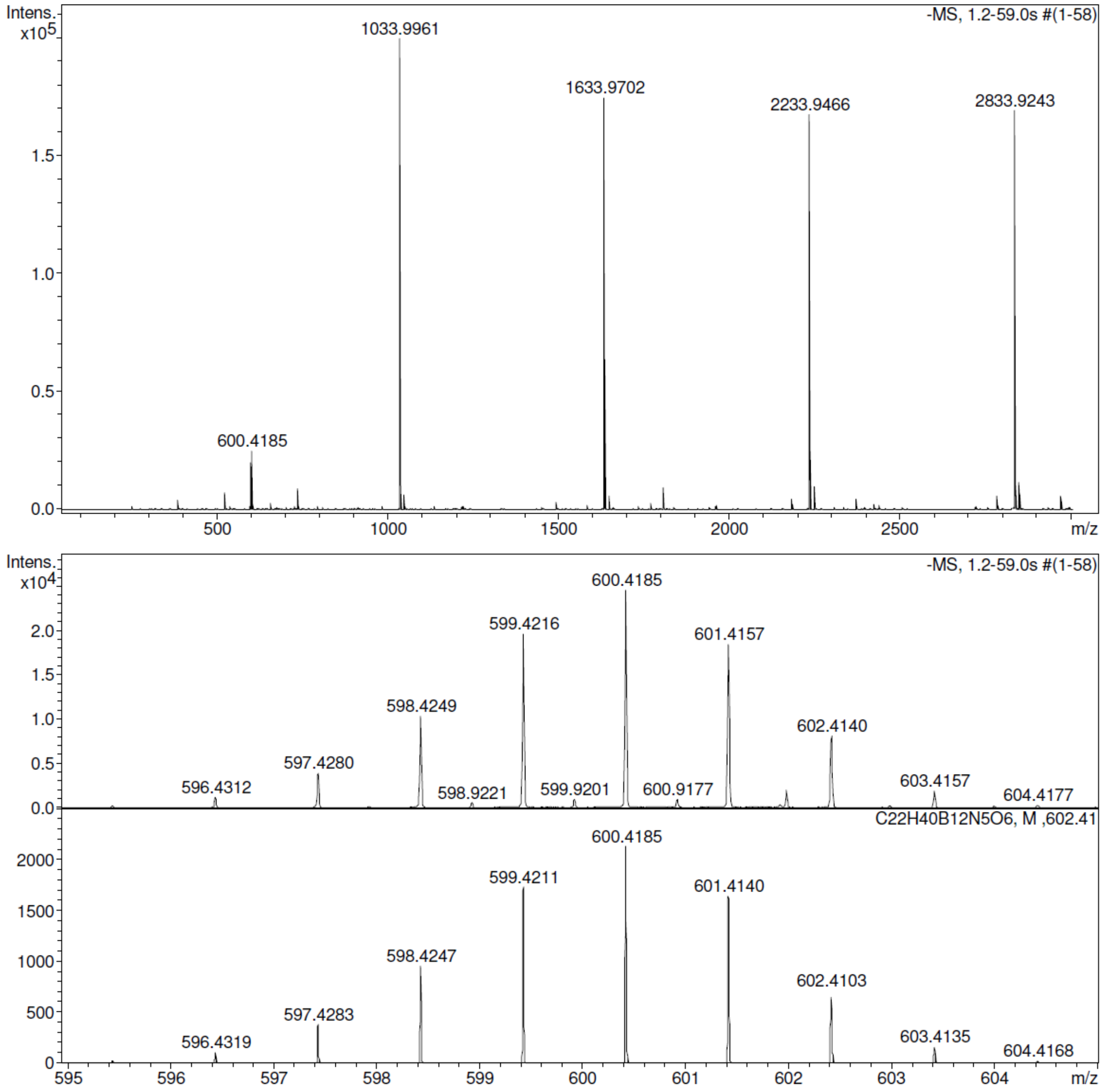

Conjugate 4 b

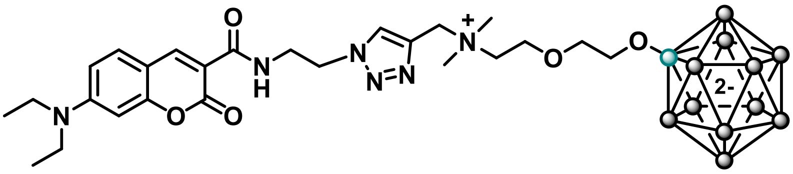

[4b]Cs

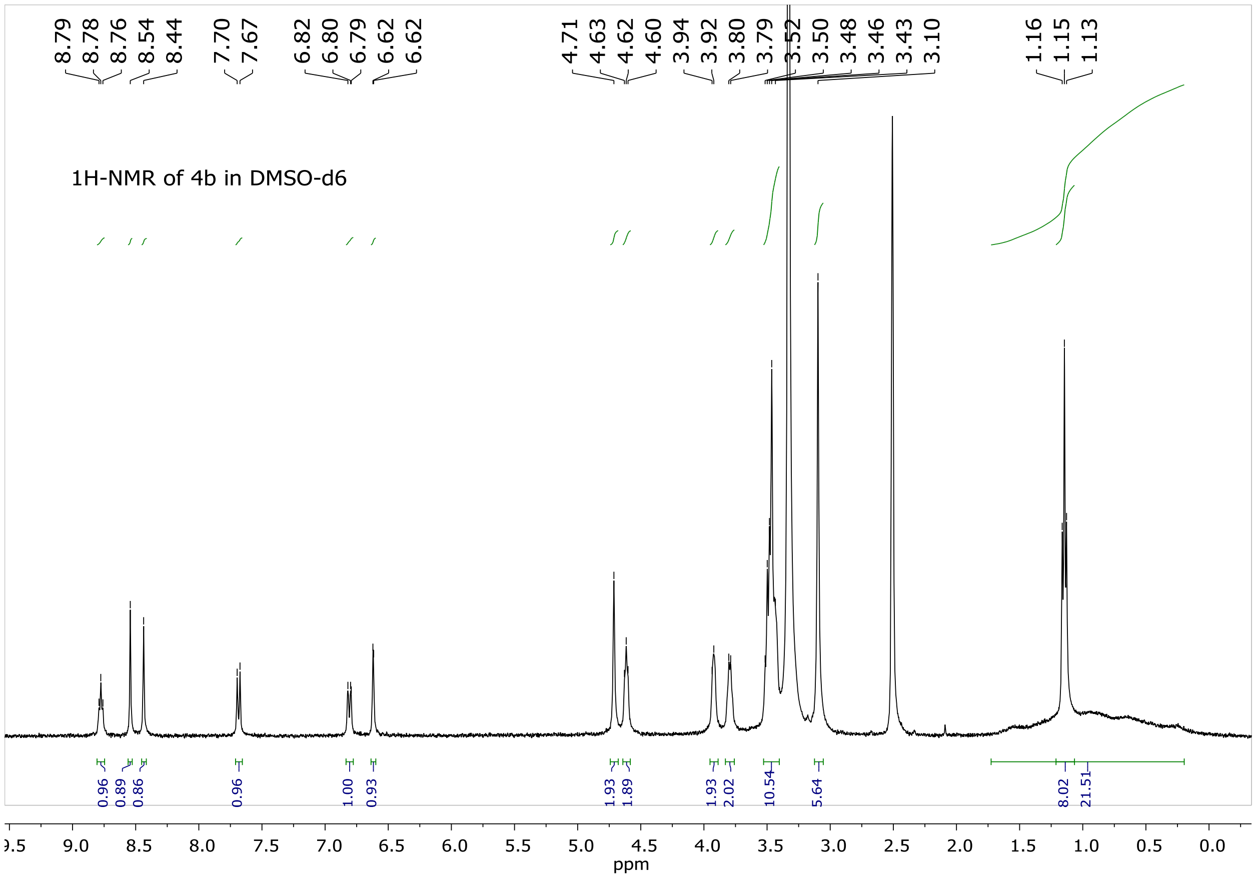
1H-NMR of 4b in DMSO-d6
8.79
8.78
8.76
8.54
8.44
7.70
7.67
6.82
6.80
6.79
6.62
6.62
4.71
4.63
4.62
4.60
3.94
3.92
3.80
3.79
3.52
3.50
3.48
3.46
3.43
3.10
1.16
1.15
1.13
0.96
0.89
0.86
0.96
1.00
0.93
1.93
1.89
1.93
2.02
10.54
5.64
8.02
21.51
ppm

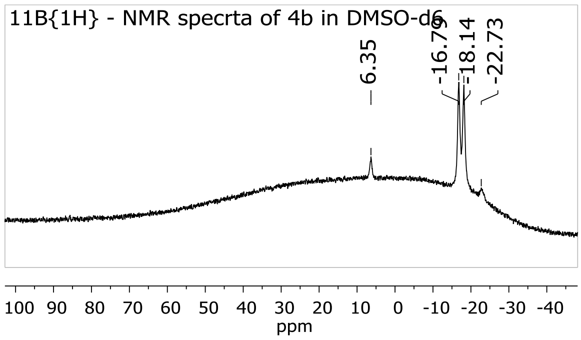
11B{1H} - NMR specrta of 4b in DMSO-d6
6.35
-16.79
-18.14
-22.73
ppm

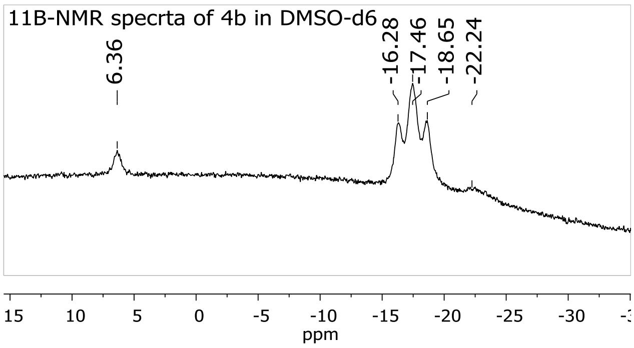
11B-NMR specrta of 4b in DMSO-d6
6.36
-16.28
-17.46
-18.65
-22.24
ppm

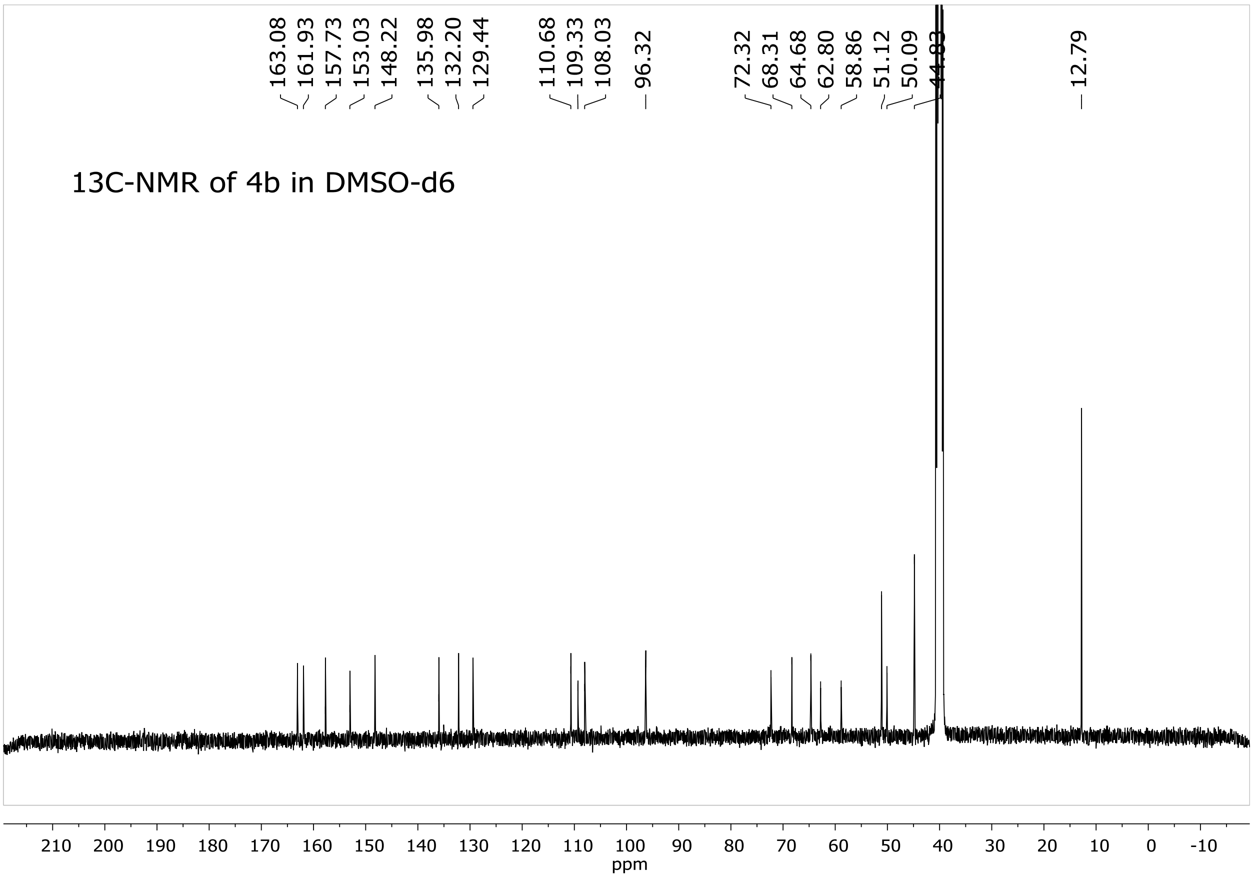
13C-NMR of 4b in DMSO-d6
163.08
161.93
157.73
153.03
148.22
135.98
132.20
129.44
110.68
109.33
108.03
96.32
72.32
68.31
64.68
62.80
58.86
51.12
50.09
44.83
12.79
210 200 190 180 170 160 150 140 130 120 110 100 90 80 70 60 50 40 30 20 10 0 -10
ppm

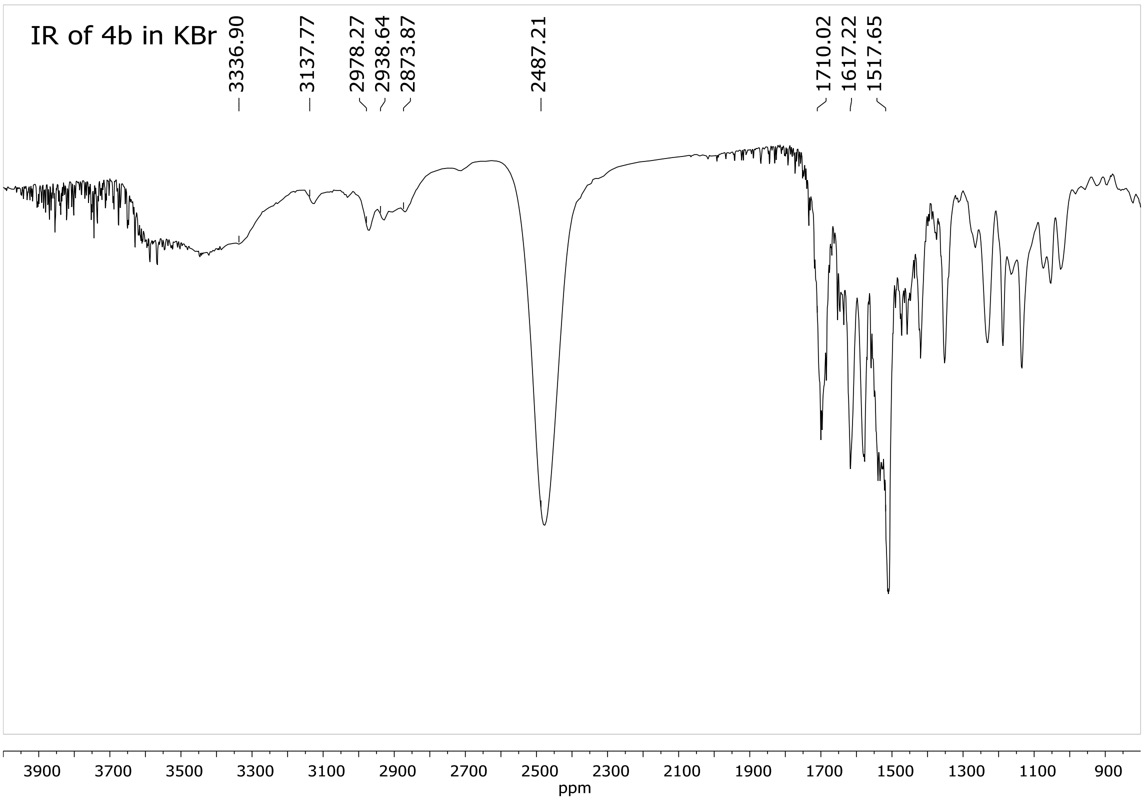
IR of 4b in KBr
3336.90
3137.77
2978.27
2938.64
2873.87
2487.21
1710.02
1617.22
1517.65
3900 3700 3500 3300 3100 2900 2700 2500 2300 2100 1900 1700 1500 1300 1100 900
ppm

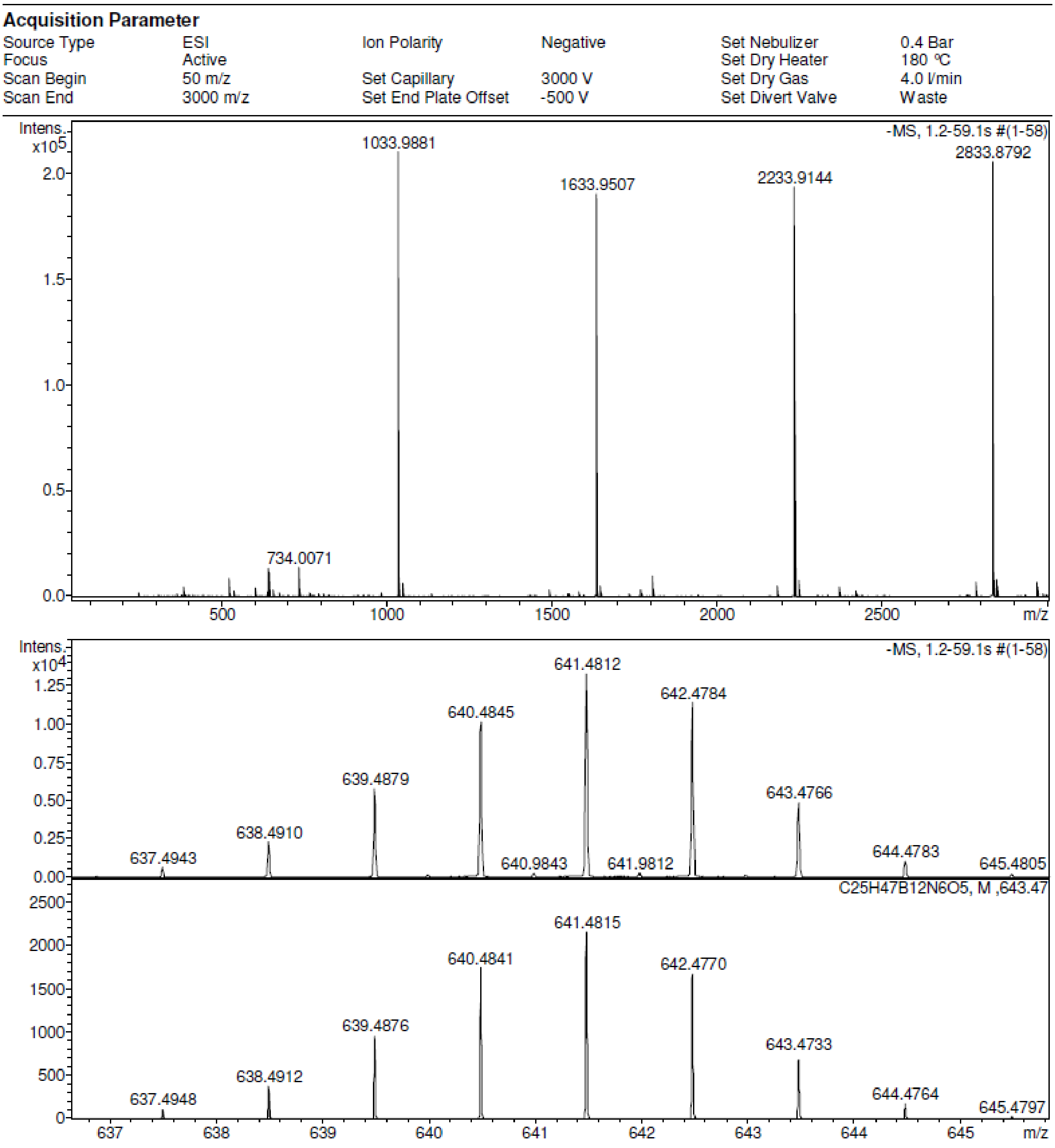

Crystallography

Table S1. Main crystallographic data and refinement details for **2a** and **2b**.

	**2a**	**2b**
Formula	$C_{13}H_{12}N_4O_4$	$C_{16}H_{19}N_5O_3$
Mass	288.27	329.36
T, K	140	140
Crystal system	Triclinic	Monoclinic
Space group	P-1	$P2_1/n$
Z	2	4

a, Å	7.1649(3)	11.4413(10)
b, Å	7.5821(4)	7.5032(7)
c, Å	12.4106(6)	19.2273(18)
a, °	95.318(2)	90
b, °	106.326(2)	103.278(3)
g, °	99.061(2)	90
V, Å^{3}	632.24(5)	1606.5(3)
d_{calc}, gЧcm^{-3}	1.514	1.362
m, cm^{-1}	1.16	0.97
F(000)	300	696
2q_{max}, °	50	50
Number of reflections measured	11190	14912
Independent reflections	3669	4663
Reflections with I>2s(I)	2091	3227
Number of parameters	238	293
R_1	0.0455	0.0452
wR_2	0.1304	0.1295
GOF	0.894	1.047
Residual electron density, eЧÅ^{-3}(d_{min}/d_{max})	0.305/-0.290	0.353/-0.213

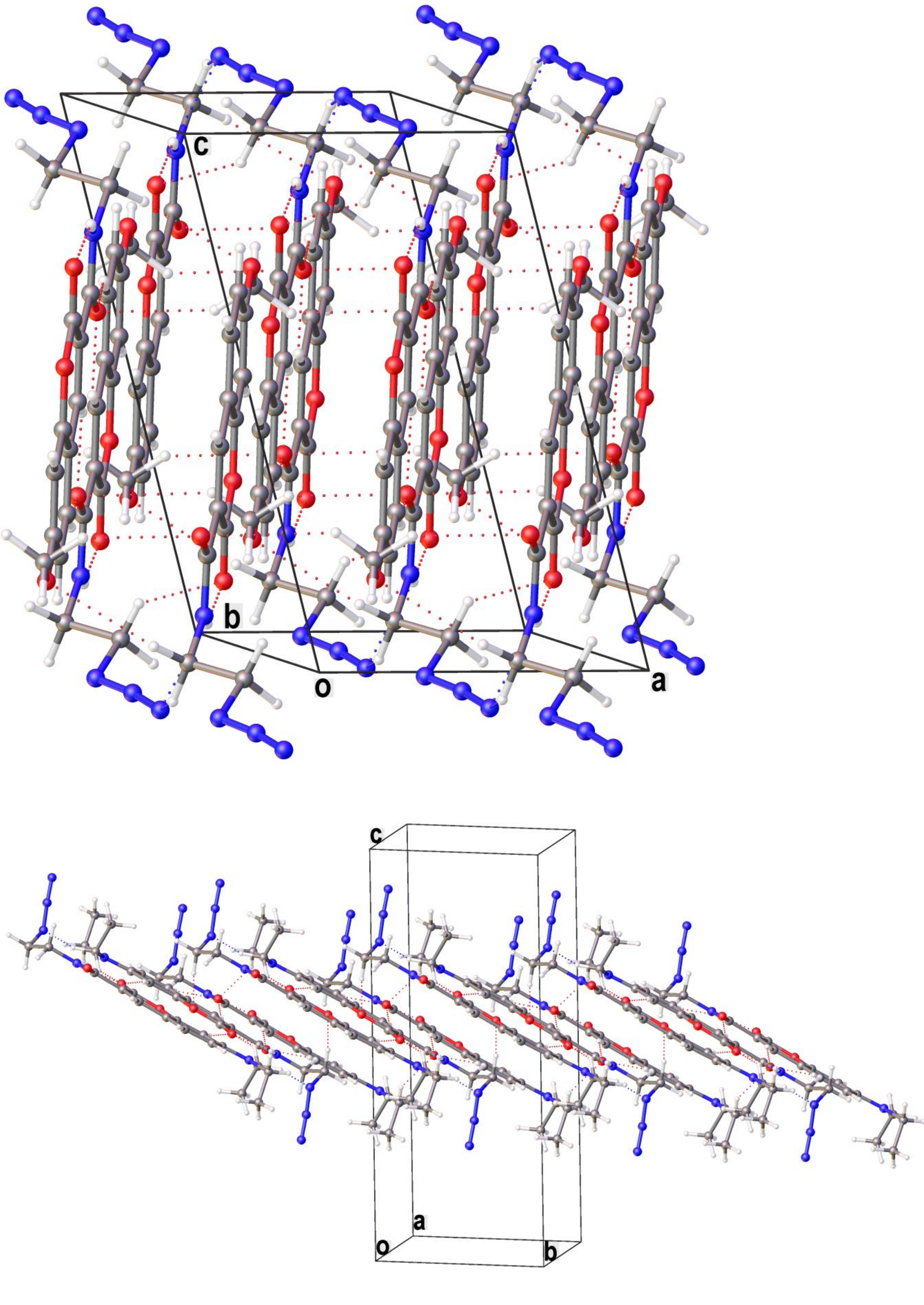

Figure S1. The layer-type crystal packing pattern in the **2a** (top) and **2b** (bottom) crystals.

Table S2. IC_{50} values for compounds **4a**, **4b** and cisplatin.

Compound	IC_{50}, μM			
	HCT116	A549	WI38	MCF7
Cisplatin	13 ± 4	13 ± 3	8 ± 3	30 ± 9
4a	>200	>200	>200	>200
4b	>200	>200	>200	>200

Results of MTT assays after a 72 h cell exposure. Shown are mean ± SD from three independent experiments
